# Transient Hypothyroidism During Lactation Arrests Myelination in the Anterior Commissure of Rats. A Magnetic Resonance Image and Electron Microscope Study

**DOI:** 10.3389/fnana.2018.00031

**Published:** 2018-04-27

**Authors:** Federico S. Lucia, Jesús Pacheco-Torres, Susana González-Granero, Santiago Canals, María-Jesús Obregón, José M. García-Verdugo, Pere Berbel

**Affiliations:** ^1^Departamento de Histología y Anatomía, Facultad de Medicina, Universidad Miguel Hernández, Sant Joan d’Alacant, Alicante, Spain; ^2^Instituto de Neurociencias de Alicante, Consejo Superior de Investigaciones Científicas, Universidad Miguel Hernández, Alicante, Spain; ^3^Laboratorio de Neurobiología Comparada, Instituto Cavanilles de Biodiversidad y Biología Evolutiva, Centro de Investigación Biomédica en Red sobre Enfermedades Neurodegenerativas, Universitat de València, Valencia, Spain; ^4^Instituto de Investigaciones Biomédicas Alberto Sols, Consejo Superior de Investigaciones Científicas, Universidad Autónoma de Madrid, Madrid, Spain

**Keywords:** limbic system, thyroid hormones, iodine diet, congenital hypothyroidism, rodent behavior, psychiatric diseases, bipolar disorders, schizophrenia

## Abstract

Thyroid hormone deficiency at early postnatal ages affects the cytoarchitecture and function of neocortical and telencephalic limbic areas, leading to impaired associative memory and in a wide spectrum of neurological and mental diseases. Neocortical areas project interhemispheric axons mostly through the corpus callosum and to a lesser extent through the anterior commissure (AC), while limbic areas mostly project through the AC and hippocampal commissures. Functional magnetic resonance data from children with late diagnosed congenital hypothyroidism and abnormal verbal memory processing, suggest altered ipsilateral and contralateral telencephalic connections. Gestational hypothyroidism affects AC development but the possible effect of transient and chronic postnatal hypothyroidism, as occurs in late diagnosed neonates with congenital hypothyroidism and in children growing up in iodine deficient areas, still remains unknown. We studied AC development using *in vivo* magnetic resonance imaging and electron microscopy in hypothyroid and control male rats. Four groups of methimazole (MMI) treated rats were studied. One group was MMI-treated from postnatal day (P) 0 to P21; some of these rats were also treated with L-thyroxine (T4) from P15 to P21, as a model for early transient hypothyroidism. Other rats were MMI-treated from P0 to P150 and from embryonic day (E) 10 to P170, as a chronic hypothyroidism group. The results were compared with age paired control rats. The normalized T2 signal using magnetic resonance image was higher in MMI-treated rats and correlated with the number and percentage of myelinated axons. Using electron microscopy, we observed decreased myelinated axon number and density in transient and chronic hypothyroid rats at P150, unmyelinated axon number increased slightly in chronic hypothyroid rats. In MMI-treated rats, the myelinated axon g-ratio and conduction velocity was similar to control rats, but with a decrease in conduction delays. These data show that early postnatal transient and chronic hypothyroidism alters AC maturation that may affect the transfer of information through the AC. The alterations cannot be recovered after delayed T4-treatment. Our data support the neurocognitive delay found in late T4-treated children with congenital hypothyroidism.

## Introduction

In humans, agenesis, dysgenesis (including ectopy) and dysfunction of the thyroid gland commonly produces congenital hypothyroidism, causing neurological and psychiatric diseases, such as intellectual disability, spasticity, and disturbances of gait and coordination (Dussault and Ruel, 1987; Rovet et al., 1992; Rovet, 1999, 2002; Brown, 2012; Clairman et al., 2015; Krude et al., 2015; Léger, 2015; Aycan et al., 2017).

Screening programs are crucial for the early detection and treatment of congenital hypothyroidism and the prevention of neurological and mental diseases in children that would result from a late diagnosis (O’Callaghan et al., 1995; Kester et al., 2004; Rovet and Simic, 2008; Williams and Hume, 2008; Willoughby et al., 2014). Some neurological deficits may persist even when diagnosis of congenital hypothyroidism is at birth (Rovet, 2002; Huo et al., 2011). Disrupted associative processing in children with early postnatal thyroid hormone insufficiency has been associated with a reduced volume and abnormal function of the hippocampus (Wheeler et al., 2015). The incidence of primary transient congenital hypothyroidism is increasing in some countries, particularly cases with milder thyroid gland dysfunction (Pearce et al., 2010; Léger et al., 2015). The reasons for this remain poorly understood, but may be related to changes in screening thresholds (Olivieri et al., 2013; Léger et al., 2015) and to gestational and early postnatal iodine deficiency (Sava et al., 1984; Köhler et al., 1996; Morreale de Escobar and Escobar del Rey, 2003; Pearce et al., 2004; Berbel et al., 2007; Walker et al., 2007; Berbel and Morreale de Escobar, 2011). In some mild iodine-deficient areas, 3.9% of women developed hypothyroidism associated with postpartum thyroiditis (Stagnaro-Green et al., 2011b), which is typically diagnosed at the sixth month postpartum (Lazarus, 2011; Stagnaro-Green et al., 2011b), that is to say at the end of the nursing period recommended by the WHO and UNICEF (Stagnaro-Green et al., 2011a). Breastfeeding children born to hypothyroid women in iodine-deficient areas, where official prevention programs and monitoring for iodine supplementation during gestation and lactation are not yet implemented, will most probably have their thyroid function compromised.

In the majority of mammals (including humans), critical processes involved in corticogenesis occur during pregnancy, while others such as the maturation and refinement of cortico-cortical connections, including the commissural connections, mostly occur postnatally (Innocenti, 1995). Telencephalic commissures in mammals are necessary for the transfer of information between cerebral hemispheres and consequently for normal telencephalic function (Innocenti and Berbel, 1991; Innocenti, 1995). Several studies have associated developmental alterations of the telencephalic commissures with psychiatric diseases such as dyslexia (Hynd et al., 1995), attention deficit-hyperactivity disorder (ADHD; Hynd et al., 1991), autism spectrum disorder (ASD; Piven et al., 1997) and schizophrenia (Innocenti et al., 2003; Guo et al., 2013; Kikinis et al., 2015). Abnormal connectivity of limbic and several neocortical areas that are contra laterally connected through the anterior commissure (AC) have been found in some of these diseases. Altered emotion, controlled by limbic areas, has been associated with bipolar disorder (LeDoux, 2000; Saxena et al., 2012; Townsend et al., 2013; Janiri et al., 2017) and ADHD (Humphreys et al., 2016). Recently, magnetic resonance imaging (MRI), has been used to observe abnormal development of interhemispheric connections in children born to women treated for hypothyroidism at different periods of pregnancy, demonstrating the contribution of maternal thyroid hormone to this process (Samadi et al., 2015). Electron microscopy (EM) data showed a significant reduction in the number of myelinated axons in the AC of gestational and postnatal hypothyroid adult rats (Berbel et al., 1994), but the effect of early transient postnatal hypothyroidism on the maturation of telencephalic commissures remains unknown.

Our aim was to study the postnatal development of the AC, from birth to adult, in transient and chronic hypothyroid rats using *in vivo* MRI and EM, and observe their recovery after delayed L-thyroxine (T4)-treatment. T_2_-ratio (T_2_r; see Materials and Methods) was correlated with quantitative EM data. The g-ratio (see Materials and Methods) and conduction velocity of myelinated axons were estimated.

## Materials and Methods

### Ethics Statement

Animal care and drug administration were performed under veterinary control according to European Union Directive 86/609/EEC with approval from the Ethics Committee of the UMH and CSIC.

### Animals and Treatments

**FIGURE 1:**
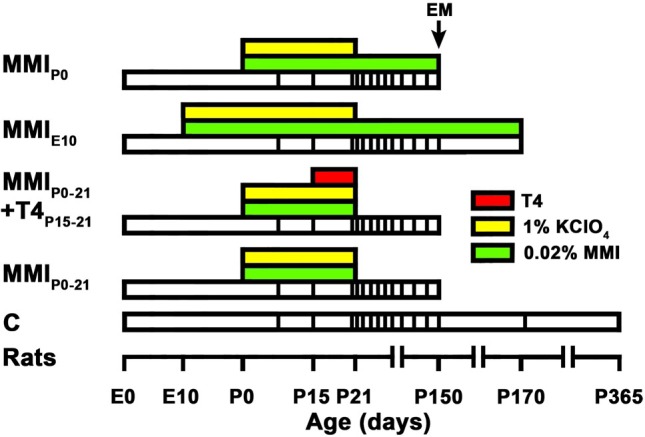
Experimental groups and treatments. Cartoon shows the experimental groups studied. Color horizontal bars show different treatments, white bars show the lifespan and at the bottom is a time scale. Vertical lines within white bars show the age of *in vivo* MRI scans. Hypothyroidism was induced by 0.02% methimazole in the drinking water (MMI; green bars). T4 (red bar) was infused subcutaneously using osmotic mini-pumps (delivering ratio: 1 μL/h/day). MMI groups were MMI-treated from the indicated age to the day of sacrifice (end of white bar). MMI_P0-21_+T4_P15-21_ group was additionally T4-treated (red bar) from P15 to P21. All MMI pups were also treated with 1% KClO_4_ (yellow bars) up to P21, in order to additionally block thyroid function during fetal and lactating periods. Four pups from two different litters per experimental group were sacrificed at P150 and studied for electron microscopy (EM). In all groups, last MRI scan was just before sacrifice at P150, except control (C) and MMI_E10_ rats not processed for EM that were additionally scanned at P170 and P365 (C rats) and at P170 (MMI_E10_).

Wistar rats were housed in temperature-controlled (22–24°C) animal quarters, with automated light and darkness cycles of 14 and 10 h. Young adult females, weighing 250–300 g, were mated with embryonic day (E) 0 counted when vaginal plug was detected. Hypothyroidism was induced by adding 0.02% methimazole (MMI, Sigma-Aldrich Co., St. Louis, MO, United States) to the drinking water. Four groups of MMI treated rats were studied (**Figure 1**). The MMI_E10_ group was treated from E10 to postnatal day (P) 150 and 170, and then sacrificed. MMI_P0-21_ and MMI_P0_ groups were treated from P0 to P21 and P150, respectively, both being sacrificed at P150. The MMI_P0-21_+T4_P15-21_ group had the same treatment as MMI_P0-21_ pups with the addition of T4 (2.4 μg/100 g of body weight/day) from P15 to P21 (sacrificed at P150). T4 was administered using osmotic mini-pumps subcutaneously with a delivering ratio of 1 μL/h/day (ALZET, model 2001; Alza Corporation, Mountain View, CA, United States). All MMI-treated rats received 1% KClO_4_ with the MMI drinking solution up to P21. In the text, MMI_P0-21_ and MMI_P0-21_+T4_P15-21_ groups are referred to collectively as transient hypothyroid rats, except when significant differences between these two groups were found. In a similar way, MMI_P0_ and MMI_E10_ groups are referred to as chronic hypothyroid rats. Control (C) rats were sacrificed at P150, P180, and P365. Four litters per group were used and culled to eight pups per litter. After weaning, dams and female pups were anesthetized by 1.5–2% isoflurane inhalation (Laboratorios Dr. Esteve, S.A., Barcelona, Spain) in O_2_ (0.9 L O_2_/min) and sacrificed by decapitation. Only male pups were used in this study.

### Determination of Total T3 and T4 Concentrations in Plasma

Under isoflurane anesthesia, eight rats per group (two per litter) were weighted and blood samples (˜1 mL) taken from the heart ventricle using a heparinized syringe, at P15, P21, and P50. The blood was spun off and plasma kept at -20°C. Following extraction and purification of the plasma samples, total thyroid hormone concentrations were obtained by radioimmunoassay (Morreale de Escobar et al., 1985).

### Electron Microscopy

Four rats per group (one per litter) were processed for EM at P150. Rats were anesthetized by 1.5–2% isoflurane inhalation (Laboratorios Dr. Esteve, S.A., Barcelona, Spain) in medical air (0.9 L/min) and perfused with saline and then fixed with 4% paraformaldehyde, 1% glutaraldehyde, 0.1M sucrose, 0.002% CaCl_2_ in 0.1M phosphate buffer (PB; pH:7.3–7.4). The brains were removed and post-fixed by immersion in the same fixative overnight, at 4°C. They were vibratome sectioned sagittally into 250 μm slices. The two most medial slices from each hemisphere were post-fixed in 2% OsO_4_ for 30 min at room temperature and stained in 2% uranyl acetate in the dark for 1 h at 4°C, dehydrated in ethanol, immersed in propylene oxide (Lab Baker, Deventry, Holland) and embedded overnight in Araldite (Durcupan, Fluka, Buchs SG, Switzerland). Ultramicrotome (Ultracut UC-6, Leica, Heidelberg, Germany) semithin sections (1.5 μm) were stained with 1% toluidine blue and ultrathin sections (60–70 nm) with lead citrate. Images were obtained with a transmission electron microscope (FEI Tecnai Spirit G2, Eindhoven, The Netherlands), using a digital camera (Morada, Soft Imaging System, Olympus).

Mid-sagittal transversal area of AC, anterior (ant-l) and posterior (post-l) limbs were measured from semi-thin sections. Evenly spaced EM photomicrographs of each AC were taken at 16,500× magnification; six in ant-l and four in post-l (see dots in the **Figure 6B** dots). Axon density, unmyelinated and myelinated axon inner diameter, and myelin thickness were obtained from plots using the Cellgraph system (Microptic S.L., Barcelona, Spain). Unmyelinated and myelinated axon number in ant-l and post-l was calculated by multiplying the mean axon density by the corresponding mid-parasagittal area measured in semi thin sections (see details in Guadaño-Ferraz et al., 1994). Myelinated axon outer diameter was calculated from axon inner diameter and myelin thickness. The ratio between inner and outer myelinated axon diameter (g-ratio), as well as the conduction velocity taken as 5.5 times the outer axon diameter (Sanders and Whitteridge, 1946; Waxman and Bennett, 1972; Innocenti, 2017) were calculated. The g-ratio indicates the percentage of myelin thickness with respect of the outer diameter of the axon since the percentage of myelin thickness = (1 – g-ratio) × 100. A higher g-ratio corresponds to a lower percentage myelin thickness (Stikov et al., 2015) and is also related to conduction velocity (Rushton, 1951).

### MRI Data Acquisition and Processing

For MRI data acquisition, rats were anesthetized in an induction chamber with 3–4% isoflurane in medical air (0.8–1 L/min) and maintained with 2% isoflurane during the scanning. Animals were placed in a custom-made MRI compatible holder with adjustable bite and ear bars, and then positioned on the magnet isocenter. Body temperature was maintained at ∼37°C through a water heat-pad and monitored using a MRI compatible control unit (MultiSens Signal conditioner, OpSens, QC, Canada). Scans were obtained with a horizontal 7 Tesla scanner with a 30 cm diameter bore (Biospec 70/30v; Bruker Medical, Ettlingen, Germany), equipped with a 675 mT/m actively shielded gradient coil (Bruker Medical; BGA 12-S) of 11.4 cm inner diameter. A ^1^H rat brain receive-only phase array coil with integrated combiner and preamplifier (no tune/no match) was used, in combination with an actively detuned transmit-only resonator (Bruker BioSpin MRI) and Paravision software (Bruker Medical).

Due to the AC antero-posterior diameter (ranging from 505 μm in P8 MMI_E10_ to 850 μm in P365 C rats) and MRI section thickness (500 μm), selection of the optimal coronal plane of study was crucial in order to avoid partial volume effects (i.e., gray matter overlap in AC). The final plane of study was determined from preliminary T2-weighted (T2w) images acquired in the three orthogonal planes using rapid acquisition relaxation enhanced sequence (RARE) with the following parameters: RARE factor 8, 15 slices, slice thickness 1 mm, field of view (FOV) 40 mm × 40 mm, matrix 256 × 256, effective echo time (TE_eff_) 56 ms, repetition time (TR) 2000 ms, 1 average for 1 min 4 s total acquisition time (Hennig and Friedburg, 1988; Perez-Cervera et al., 2018). Using these anatomical images, final coronal MRI images were acquired using RARE sequence with the following parameters: RARE factor 8, 25 slices, slice thickness 0.5 mm, FOV 20 mm × 20 mm, matrix 200×200 (voxel size 100 μm × 100 μm × 500 μm), TE_eff_ 56 ms, TR 3,728 ms, four averages for 12 min 26 s total acquisition time.

Magnetic resonance imaging images were obtained from eight rats per group (two rats per litter) at ages from P8 to P365 (vertical lines within bars in **Figure 1** and Supplementary Table 2) and analyzed using ImageJ (National Institute of Health, Bethesda, MD, United States). The region of interest (ROI) of the AC included the medial portion at ±0.7 mm from of the midline (**Figure 3A**, vertical white lines in P150C). The T2 ratio (T_2_r) was calculated as the ratio between the signal intensity of T2w in the ROI to that of T2w in a ROI in the lateral ventricle cerebrospinal fluid. T_2_r was obtained in order to compare T2w signals between rats and experimental groups.

We measured contralateral distances between selected homotopic contralateral areas projecting through the AC using consecutive MRI scans, specifically the olfactory tubercle and the anterior piriform cortex projecting through ant-l, and perirhinal and posterior piriform cortices and lateral amygdaloid nucleus through post-l (including the stria terminalis; see schema in Guadaño-Ferraz et al., 1994; **Figure 12**). Due to MMI brain shrinkage not found in C rats, anterior and posterior anatomical landmarks were used to identify equivalent projecting areas (Supplementary Figure 1). The anterior landmark was the point where the antero-lateral branch of the ant-l begins to appear transversally cut (at 1.89 mm from Bregma in C rats; Paxinos et al., 2015). At this level, olfactory tubercle and anterior piriform cortex were present in MMI and C rats. The posterior landmark was the point where lateral and medial habenular nuclei appeared darker than the adjacent neuropil (at -3.36 mm from Bregma in C rats: Paxinos et al., 2015). At this level, perirhinal and posterior piriform cortices and lateral amygdaloid nucleus were present in both MMI and C rats. Contralateral distances were measured between the central zones of the lateral amygdaloid nuclei and between mid-zones of pial surfaces in other areas. Taking into account the 500 μm thickness of the MRI scans, distance measurements may have an antero-posterior error of about 250 μm per hemisphere. Conduction delay between homotopic areas was calculated from the mean distance between areas and conduction velocity.

### Statistical Analysis

For statistical analysis, we used SYSTAT software (Systat Software, Inc., Chicago, IL, United States). Mean frequency distributions of MRI and EM data were analyzed using two-way ANOVA followed by either Tukey’s (equal variances) or Games–Howell’s (unequal variances) tests to identify significant differences (*P* ≤ 0.05) between means among age and experimental groups. The plasma concentration of thyroid hormones was analyzed using one-way ANOVA followed by either Tukey’s test or the Student–Newman–Keuls method. Correlation coefficient (*R*^2^) refers to the Pearson’s product-moment correlation coefficient.

## Results

### Body Weight and Thyroid Hormone Levels

**FIGURE 2:**
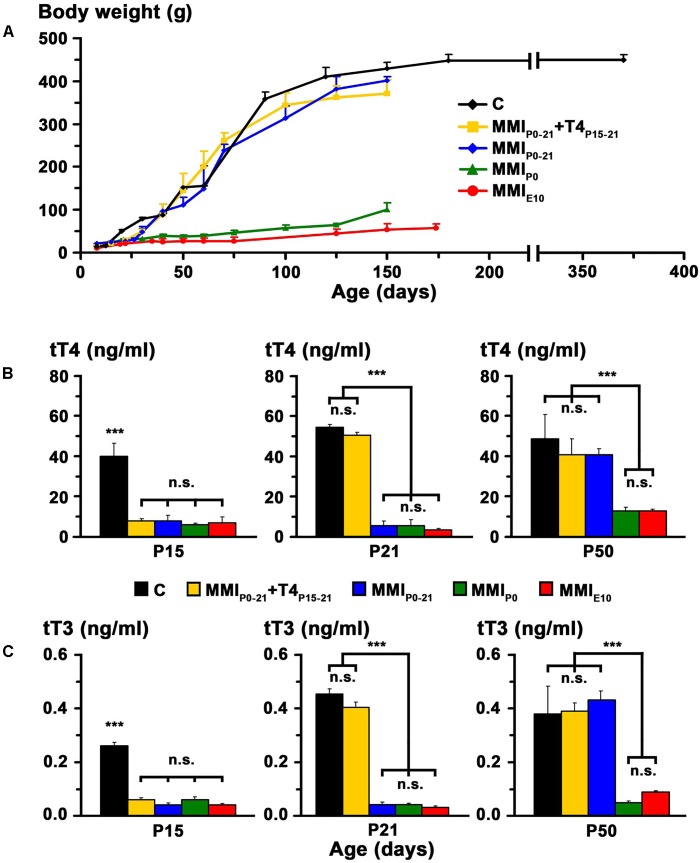
Body weight and plasma concentration levels of thyroid hormone (TH). Changes of body weight with age in C and MMI rats **(A)**. Note the arrested growth of chronic hypothyroid pups. Bar histograms **(B,C)** show the total plasma concentrations of T4 (tT4) and T3 (tT3) at the indicated ages. TH plasma concentrations were recovered in transient hypothyroid rats at P50. Bars: mean ± SD. n.s., non-significant differences. Significant differences: ^∗∗∗^*P* ≤ 0.001 (*n* = 8–11 rats per group).

In transient hypothyroid and control rats, body weight rapidly increased to 400 ± 20 g by P150 and then more slowly to 446 ± 13 g in C rats at P365. Weight gain was significantly lower (*P* < 0.001) in chronic hypothyroid rats with an average value of 75 ± 25 g at P150 (**Figure 2A**).

At P15, plasma concentration levels of total T4 (tT4) and total T3 (tT3) in MMI rats had an average value of 7.2 and 0.05 ng/ml respectively, and were significantly lower (*P* < 0.001) than the control averages of 40.1 ng tT4/ml and 0.26 ng tT3/ml. Normal levels of tT4 and tT3 were reached at P21 in MMI_P0-21_+T4_P15-21_ rats and at P50 in MMI_P0-21_ rats. Levels of tT4 and tT3 in P50 chronic hypothyroid rats, was significantly lower (*P* < 0.001) than in transient hypothyroid and C rats of the same age (**Figures 2B,C** and Supplementary Table 1).

### MRI Data

**FIGURE 3:**
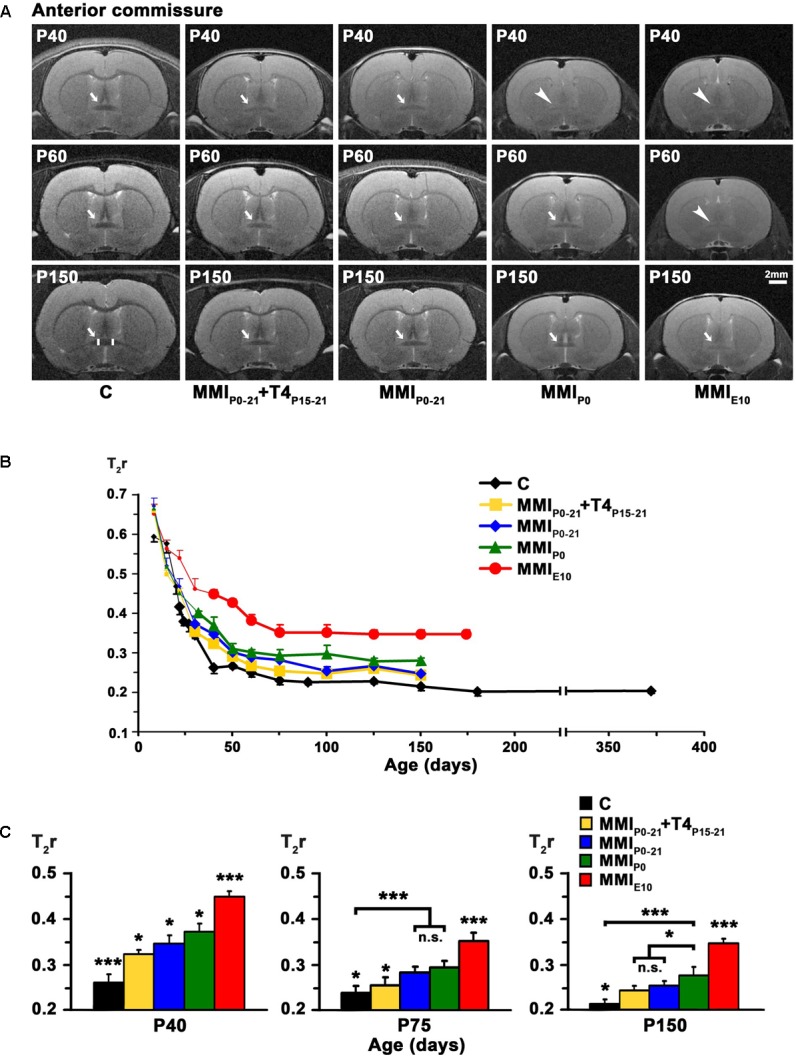
MRI images and T_2_r of the AC at postnatal ages. At all ages, T2-weighted images **(A)** of the AC (arrows and arrowheads) of MMI rats were less contrasted than in controls (arrowheads point undistinguishable AC). It remained hardly visible in chronic hypothyroid rats at P150 (arrow). A region of interest (ROI; area between vertical white bars) of the AC is shown in P150 C rats. Changes of T_2_r at postnatal ages **(B)**; bold dots show T_2_r values when AC was darker than the adjacent neuropil. In C rats, T_2_r decreased rapidly from P8 to P50 and then more slowly. In MMI rats, T_2_r values showed a similar trend but remained higher than in C rats. Bar histograms **(C)** show that at all ages, T_2_r was significantly higher in MMI than in C rats. Bars: mean ± SD. n.s., non-significant differences. Significant differences: ^∗^*P* ≤ 0.05 and ^∗∗∗^*P* ≤ 0.001 (*n* = 8 rats per group).

Anterior commissure appeared lighter than the adjacent neuropil in MRI scans of both MMI and C rats at an early age, with the AC being hardly distinguishable. T_2_r progressively decreased with age and at T_2_r ≤ 0.45 which occurred at P22 in C rats and at older ages in MMI rats, AC darkened and showed increased border definition (arrows and bold lines in **Figures 3A,B** and Supplementary Table 2). The AC remained hardly distinguishable in MMI_P0_ at P40 and in MMI_E10_ rats at P60 (arrowheads in **Figure 3A**). In C rats, T_2_r decreased rapidly from P8 (T_2_r = 0.59 ± 0.01) to P40 (T_2_r = 0.26 ± 0.02) and then more slowly, reaching T_2_r = 0.21 ± 0.01 at P365. MMI rat, T_2_r also decreased rapidly from P8 to P40 but remained higher than controls (**Figure 3B** and Supplementary Table 2). At P40, significant T_2_r differences (*P* < 0.05) were found between MMI_P0-21_+T4_P15-21_, MMI_P0-21_ and MMI_P0_ rats, which showed even greater differences (*P* < 0.001) with respect to C and MMI_E10_ rats (**Figure 3C** and Supplementary Table 2). At P150, T_2_r was similar in transient hypothyroid rats but not to C (*P* < 0.05), MMI_P0_ (*P* < 0.05) and MMI_E10_ (*P* < 0.001) rats (**Figure 3C** and Supplementary Table 2). These data show that AC maturation might be delayed, at least until P150, in transient and chronic hypothyroid rats.

**FIGURE 4:**
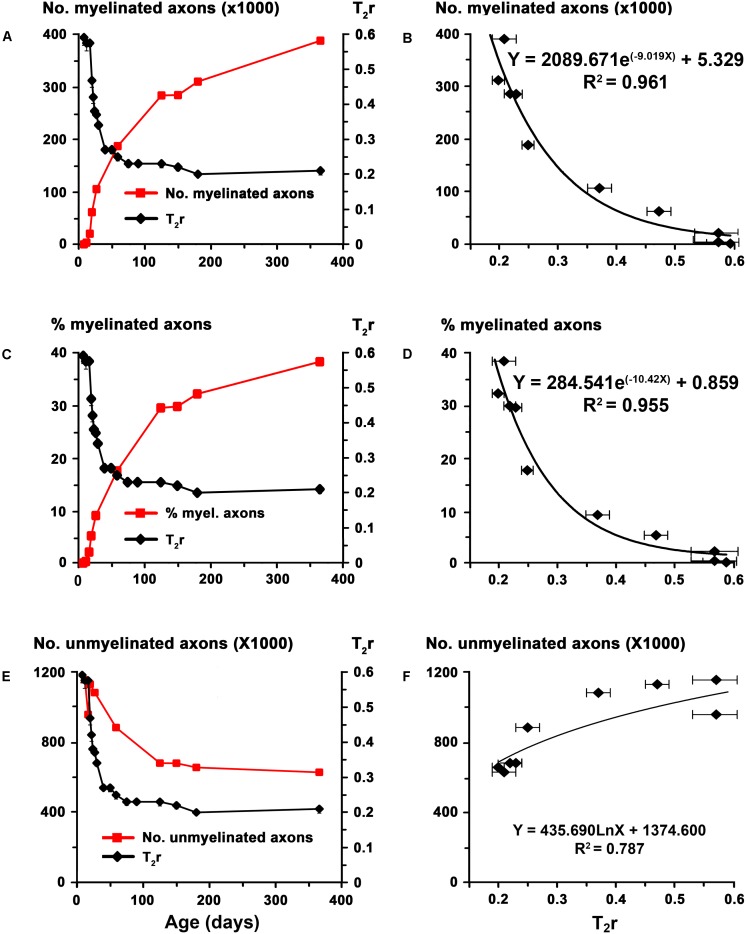
Correlation of T_2_r with EM data in the AC at postnatal ages. Line charts **(A,C,E)** show myelinated axon number and percentage, unmyelinated axon number in C rats [red lines; EM data in **(A,C,E)** are from Berbel et al., 1994] and T_2_r (black lines) at certain points of postnatal age. Regression functions **(B,D,F)** between EM data and T_2_r. High correlations were found between T_2_r and myelinated axon number (**B**; *R*^2^ = 0.961) and percentage (**D**; *R*^2^ = 0.955), while unmyelinated axon number (**F**; *R*^2^ = 0.787) correlated poorly with T_2_r.

**FIGURE 5:**
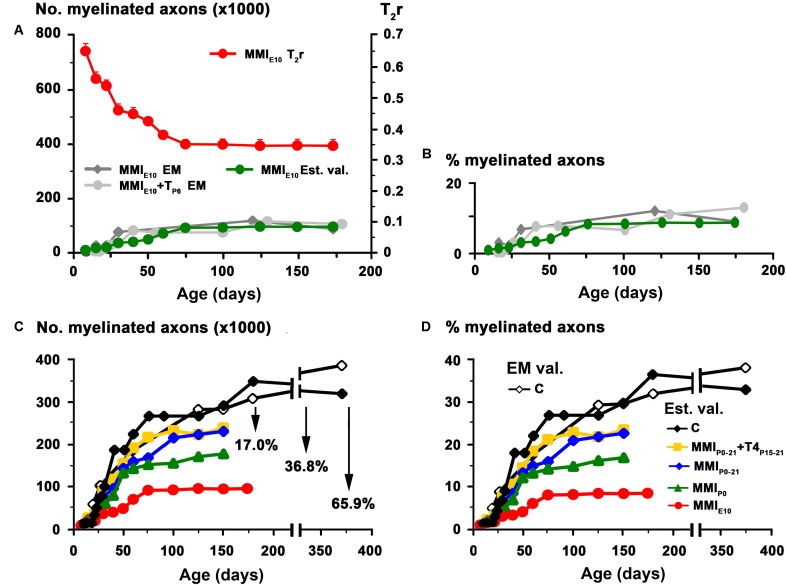
Estimated values for MMI treated rats. Estimated values (green dots) of myelinated axon number **(A)** and percentage **(B)** for MMI_E10_ rats obtained using regression functions shown in **Figures 4B,D**. These values were similar to those previously published in EM studies (MMI_E10_ – gray dots – and MMI_E10_+T_P6_ – dark-gray dots-groups in Berbel et al., 1994). These regression functions were used to estimate myelinated axon number **(C)** and percentage **(D)** at postnatal ages in C, transient and chronic hypothyroid rats. At P150, estimated values (Est. val.) of myelinated axon number and percentage of C rats was similar to published values (EM val.; Berbel et al., 1994). Estimated values of myelinated axon number **(C)** for transient hypothyroid (on average, 17%), MMI_P0_ (38.8%), and MMI_E10_ (65.9%) decreased with respect to C rats. A similar decrease was found for the estimated myelinated axon percentage **(D)**.

The biological significance of T_2_r was explored by correlating these values with quantitative EM data, and generating the corresponding regression functions. Using the EM data from the AC in C rats at different postnatal ages (Berbel et al., 1994), we found a significant correlation between T_2_r and myelinated axon number (*R*^2^ = 0.96; **Figures 4A,B**) and percentage (*R*^2^ = 0.96; **Figures 4C,D**). Unmyelinated axon number (*R*^2^ = 0.79; **Figures 4E,F**) correlated poorly with T_2_r. In order to validate these correlations, we used them to estimate myelinated axon number and percentage in MMI_E10_ rats and compare the values obtained with those published in the literature (**Figures 4A,B**). No significant differences were found between estimated MMI_E10_ values and those in previously published EM studies (MMI_E10_ and MMI_E10_+T_P6_ groups in Berbel et al. (1994) (**Figures 5A,B**). Consequently, these regression functions were used to estimate myelinated axon number and percentage for C, transient and chronic hypothyroid rats. At P150, these estimations indicated a significant decrease in myelinated axon number in transient hypothyroid (17.0%), MMI_P0_ (36.8%) and MMI_E10_ (65.9%) rats with respect to C rats (**Figure 5C**). A similar decrease was also found in myelinated axon percentage with respect to C rats (**Figure 5D**).

### EM Study

**FIGURE 6:**
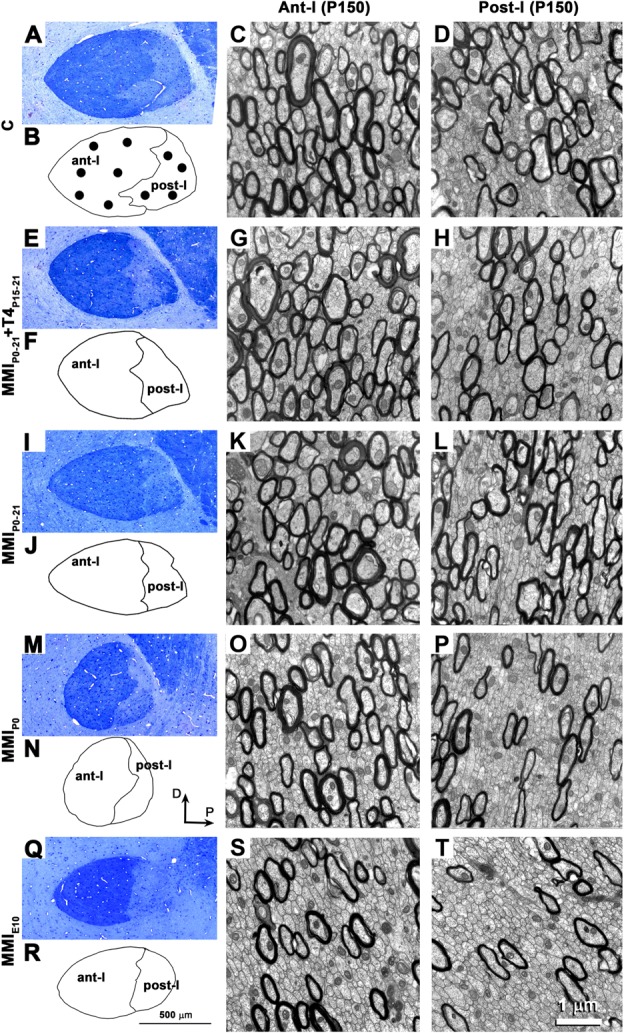
Optical and electron microscope (EM) structure of the AC at P150. Semi-thin sections showing the AC **(A,E,I,M,Q)**. Outlines show the AC’s boundaries with the neuropil and between limbs **(B,F,J,N,R)**. Note the decreased midsagittal transversal AC area in MMI compared to C rats. EM images show the ultrastructure of ant-l **(C,G,K,O,S)** and post-l **(D,H,L,P,T)**. Note the decreased myelinated axon density in post-l compared to ant-l. In all groups, the myelin thickness was similar. Dots **(B)** show the approximate sites where EM photomicrographs were taken. Same magnification for optical (bar in **R**) and EM micrographs (bar in **T**).

The ultrastructure of neuron, glial cell somata and commissural axons in ant-l and post-l (**Figure 6**) was similar to that previously described in C (Sturrock, 1975, 1977; Guadaño-Ferraz et al., 1994; Larriva-Sahd et al., 2002) and MMI-treated (Guadaño-Ferraz et al., 1994) rats. The increased myelinated axon density in ant-l compared to post-l in each group (compare **Figures 6C,G,K,O,S** with **Figures 6D,H,L,P,T**, respectively), and the decreased myelinated axon density in ant-l and post-l of chronic hypothyroid compared to transient hypothyroid and C rats (compare **Figures 6O,S** with **Figures 6C,G,K**, and **Figures 6P,T** with **Figures 6D,H,L**, respectively) is noteworthy. The AC mid-sagittal area was 201,428 ± 10,377 μm^2^ in C rats and significantly decreased to 174,204 ± 4,346 μm^2^ in transient hypothyroid (*P* < 0.05), and to 123,988 ± 7,842 μm^2^ in chronic hypothyroid rats (*P* < 0.001), representing a 13.5% and 38.4% reduction, respectively with respect to C rats. Ant-l area in C and MMI rats was, on average, 72.3% of the total AC area (left outlines in **Figure 6**).

**FIGURE 7:**
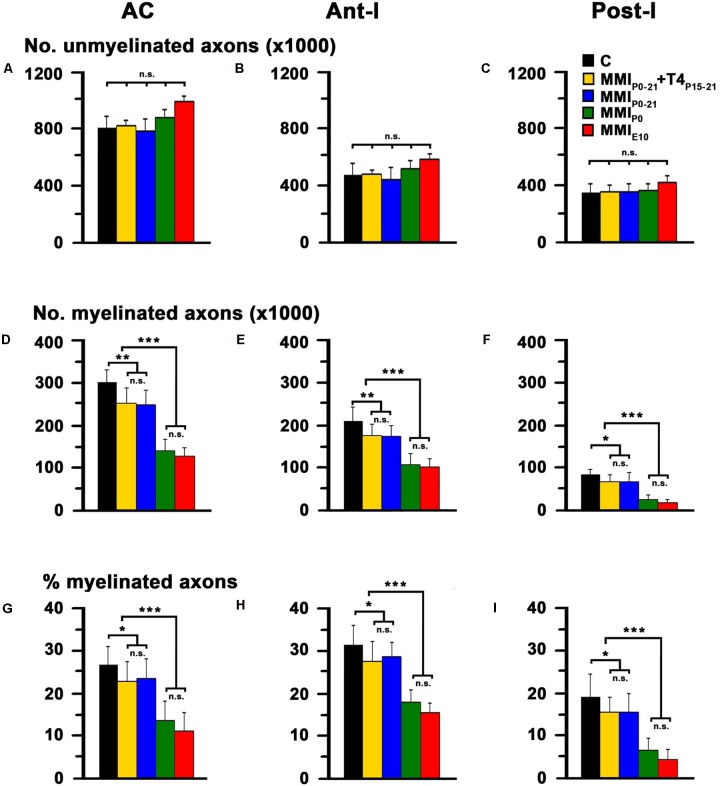
Electron microscope (EM) quantitative data of the AC at P150. Bar histograms show unmyelinated axon number **(A–C)**, and myelinated axon number **(D–F)** and percentage **(G–I)** in AC, ant-l and post-l. The total unmyelinated axon number in AC, ant-l and post-l was similar between MMI and C rats **(A–C)**. In contrast, myelinated axon number **(D–F)** and percentage **(G–I)** in AC, ant-l and post-l decreased significantly in MMI with respect to C rats. Bars: mean ± SD. n.s., non-significant differences. Significant differences: ^∗^*P* ≤ 0.05 and ^∗∗∗^*P* ≤ 0.001 (*n* = 4 rats per group).

**FIGURE 8:**
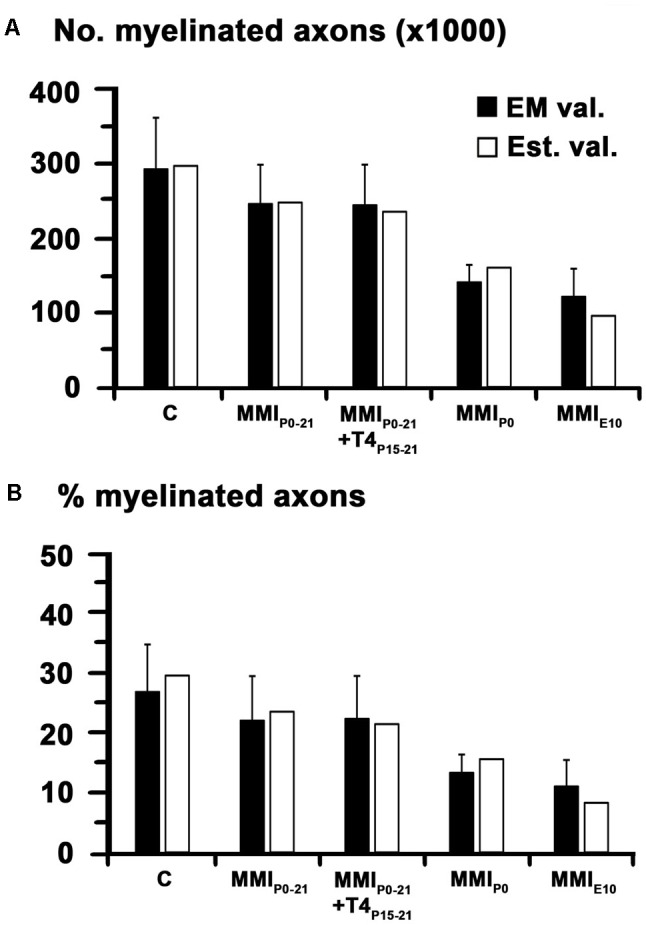
Comparison between T_2_r-estimated and electron microscope (EM)-observed values. Bar histograms show EM (EM val.) and T_2_r-estimated values (Est. val.) of myelinated axon number **(A)** and percentage **(B)**. The myelinated axon number and percentage in AC was similar to that estimated using regression functions for C rats. Black bars: means ± SD.

In agreement with previous data, the total AC axon number was similar in C and MMI rats at P150, ranging from 1,009,160 axons in MMI_P0_ to 1,114,058 axons in MMI_E10_ rats (1,088,607 in C rats; **Table 1**). However, myelinated axon number decreased significantly in transient 16.9% (*P* < 0.05) and chronic 55.2% (*P* < 0.001) hypothyroid rats, with respect to C rats (**Figure 7** and **Table 1**). In all experimental groups, ant-l myelinated axon number was higher than in post-l (72.3% axons in C, 73.2% in transient and 82.5% in chronic hypothyroid rats; **Figures 7E,F** and **Table 1**). Similar differences were found in myelinated axon percentage (**Figures 7H,I** and **Table 1**). AC myelinated axon number and percentage was similar to that estimated using regression functions for C rats. There were 288,765 myelinated axons in C compared to the 292,645 estimated, 239,707 transient compared to 234,880 estimated and 129,231 chronic hypothyroid compared to 133,427 estimated (**Figure 8A**), giving the following percentages of real and estimated myelinated axons: 26.9 and 29.6% in C; 23.2 and 23.0% in transient; 12.4 and 12.3% in chronic hypothyroid rats (**Figure 8B**).

**Table 1:** Axon number and percentage at P150.

	C	MMI_P0-21_+T4_P15-21_	MMI_P0-21_	MMI_P0_	MMI_E10_
**Ant-l**					
Unmyel. ax. no. (×10^3^)	462.2 ± 86.4	468.6 ± 71.9	438.1 ± 80.6	509.9 ± 56.3	576.5 ± 34.5
Myel. ax. no. (×10^3^)	208.8 ± 42.1	176.7 ± 34.7	174.3 ± 32.5	110.0 ± 25.0	104.6 ± 18.6
Myel. ax. %	31.1 ± 4.7	26.5 ± 4.6	26.9 ± 3.2	17.7 ± 3.0	15.4 ± 2.3
**Post-l**					
Unmyel. ax. no. (×10^3^)	337.6 ± 69.9	350.6 ± 48.0	347.4 ± 54.9	363.9 ± 38.6	414.6 ± 52.6
Myel. ax. no. (×10^3^)	80.0 ± 12.1	64.5 ± 15.9	63.9 ± 21.2	25.4 ± 10.4	18.5 ± 5.5
Myel. ax. %	19.2 ± 5.4	16.0 ± 3.5	16.0 ± 4.4	6.5 ± 2.8	4.3 ± 2.5
**AC**					
Unmyel. ax. no. (×10^3^)	799.8 ± 98.7	819.3 ± 85.2	785.6 ± 73.5	873.8 ± 81.9	991.1 ± 62.2
Myel. ax. no. (×10^3^)	288.8 ± 72.3	241.2 ± 62.8	238.2 ± 60.9	135.4 ± 46.5	123.1 ± 44.5
Myel. ax. %	26.5 ± 8.2	22.9 ± 6.7	23.4 ± 6.7	13.4 ± 5.6	11.1 ± 5.4

*Data are mean ± SD. Four rats per group. Ax, axon; Unmyel, unmyelinated; Myel, myelinated; Ant-l, anterior limb; Post-l, posterior limb; AC, anterior commissure.*

**FIGURE 9:**
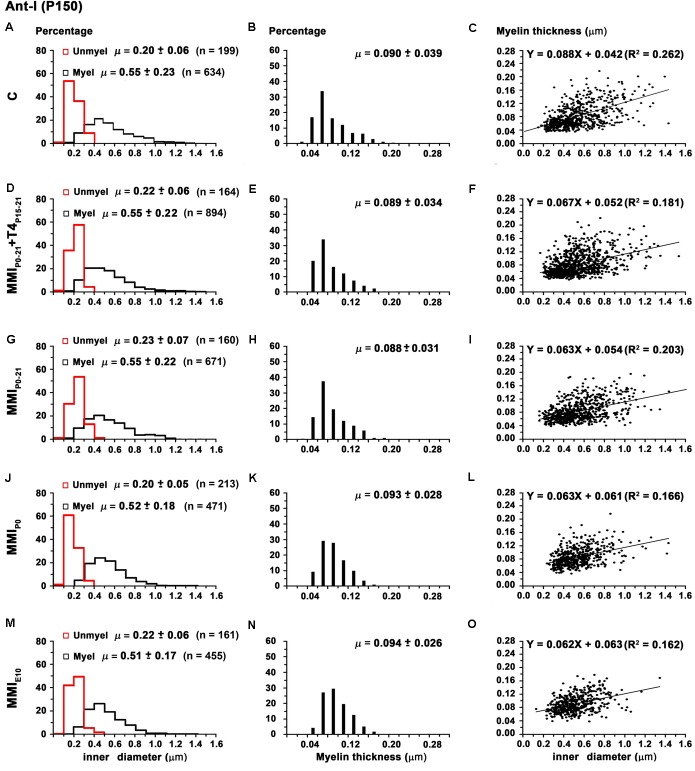
Distribution of myelinated axon diameter and myelin thickness in ant-l at P150. Histograms **(A,D,G,J,M)** show unmyelinated and myelinated axon diameter distribution in ant-l. Mean unmyelinated axon diameter was similar between MMI and C rats, while myelinated axon diameter decreased (*P* < 0.05) in chronic hypothyroid rats. Histograms **(B,E,H,K,N)** show myelin thickness distribution. The mean myelin thickness increased (*P* < 0.05) in chronic hypothyroid respect to transient hypothyroid and C rats. Plots **(C,F,I,L,O)** show the correlation between myelinated axon inner diameter and myelin thickness. Note the poor correlation found (*R*^2^ range 0.062-0.162). Nevertheless, the slope of the regression function was higher in C (5.0°) than in MMI (on average, 3.6 ± 0.1°) rats. Correlation coefficient (*R*^2^), number of axons (*n*) and means (μ = mean ± SD) are indicated.

**FIGURE 10:**
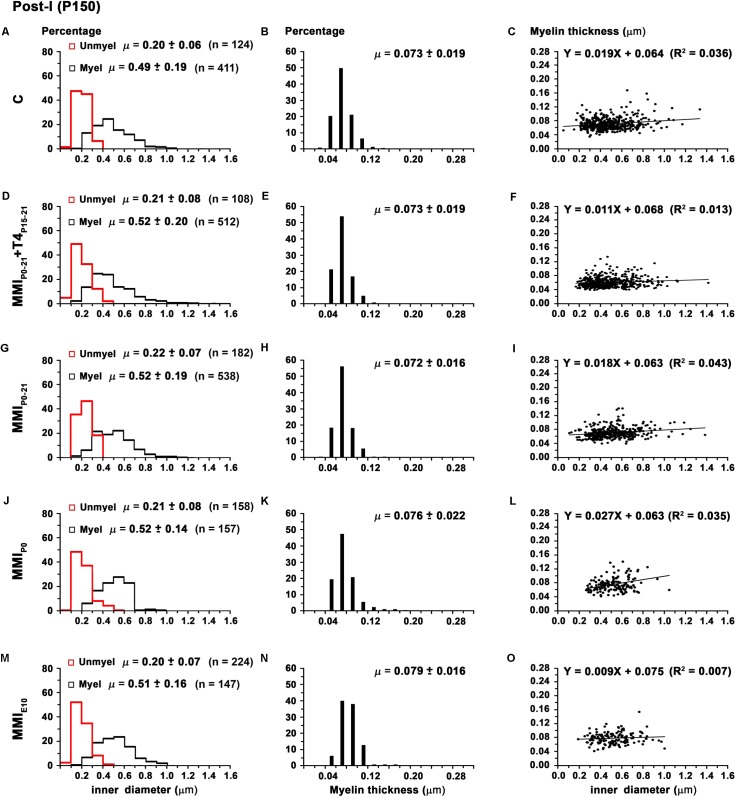
Distribution of myelinated axon diameter and myelin thickness in post-l at P150. Histograms **(A,D,G,J,M)** show unmyelinated and myelinated axon diameter distribution in post-l. Unmyelinated and myelinated axon diameter was similar between C and MMI rats. Histograms **(B,E,H,K,N)** show myelin thickness distribution. The mean myelin thickness increased (*P* < 0.05) in chronic hypothyroid respect to transient hypothyroid and C rats. Plots **(C,F,I,L,O)** show the correlation between myelinated axon inner diameter and myelin thickness. Note the poor correlation found (*R*^2^ range 0.007–0.043). The slope of the regression function in C and MMI rats was very low (on average, 0.9 ± 0.4°). Correlation coefficient (*R*^2^), number of axons (*n*) and means (μ = mean ± SD) are indicated.

There were no significant differences in average unmyelinated and myelinated axon inner diameter in the AC of C and MMI rats (0.21 ± 0.07 and 0.53 ± 0.20 μm, respectively; **Table 2**). In ant-l, unmyelinated axon diameter was similar between groups (on average, 0.21 ± 0.06 μm). Myelinated axon inner diameter decreased (*P* < 0.05) in chronic hypothyroid rats (0.52 ± 0.17 μm) compared to transient hypothyroid and C rats (0.55 ± 0.22 μm; **Figures 9A,D,G,J,M** and **Table 2**). Post-l average axon diameter was similar in C and MMI rats for both unmyelinated (0.21 ± 0.07 μm) and myelinated (0.51 ± 0.18 μm) (**Figures 10A,D,G,J,M** and **Table 2**). In ant-l and post-l of C rats, 3.5% of the myelinated axons had an inner diameter greater than 1.0 μm, whilst in transient rats it was 1.6% and in chronic hypothyroid 0.9% (**Figures 9A,D,G,J,M**, **10A,D,G,J,M**).

**Table 2:** Axon diameter (μm), myelin thickness (μm), g-ratio and conduction velocity (m/s) at P150.

	C	MMI_P0-21_+T4_P15-21_	MMI_P0-21_	MMI_P0_	MMI_E10_
**AC**					
Unmyel axon diam. (median)	0.20 ± 0.06 (0.20)	0.21 ± 0.07 (0.21)	0.23 ± 0.07 (0.23)	0.20 ± 0.07 (0.19)	0.21 ± 0.07 (0.20)
Myel axon inner diam. (median)	0.53 ± 0.21 (0.49)	0.54 ± 0.21 (0.50)	0.53 ± 0.21 (0.51)	0.52 ± 0.17 (0.50)	0.51 ± 0.17 (0.49)
Myelin thickness (median)	0.083 ± 0.033 (0.073)	0.083 ± 0.031 (0.074)	0.081 ± 0.026 (0.073)	0.089 ± 0.028 (0.084)	0.090 ± 0.025 (0.085)
g-Ratio (median)	0.75 ± 0.08 (0.76)	0.75 ± 0.08 (0.76)	0.76 ± 0.07 (0.76)	0.74 ± 0.06 (0.74)	0.73 ± 0.07 (0.73)
Conduction velocity (median)	3.80 ± 1.38 (3.51)	3.87 ± 1.32 (3.62)	3.83 ± 1.29 (3.66)	3.84 ± 1.12 (3.74)	3.84 ± 1.04 (3.61)
**Ant-l**					
Unmyel axon diam. (median)	0.20 ± 0.06 (0.19)	0.22 ± 0.06 (0.22)	0.23 ± 0.07 (0.23)	0.20 ± 0.05 (0.19)	0.22 ± 0.06 (0.22)
Myel axon inner diam. (median)	0.55 ± 0.23 (0.50)	0.55 ± 0.22 (0.51)	0.55 ± 0.22 (0.51)	0.52 ± 0.18 (0.49)	0.51 ± 0.17 (0.49)
Myelin thickness (median)	0.090 ± 0.039 (0.078)	0.089 ± 0.034 (0.078)	0.088 ± 0.031 (0.078)	0.093 ± 0.028 (0.085)	0.094 ± 0.094 (0.092)
g-Ratio (median)	0.75 ± 0.07 (0.75)	0.75 ± 0.07 (0.75)	0.75 ± 0.07 (0.76)	0.73 ± 0.06 (0.73)	0.72 ± 0,07 (0.72)
Conduction velocity (median)	3.99 ± 1.51 (3.67)	3.98 ± 1.41 (3.73)	3.98 ± 1.41 (3.74)	3.90 ± 1.15 (3.74)	3.85 ± 1.08 (3.65)
**Post-l**					
Unmyel axon diam. (median)	0.20 ± 0.06 (0.20)	0.21 ± 0.08 (0.21)	0.22 ± 0.07 (0.23)	0.21 ± 0.08 (0.22)	0.20 ± 0.07 (0.20)
Myel axon inner diam. (median)	0.49 ± 0.19 (0.46)	0.52 ± 0.20 (0.49)	0.52 ± 0.19 (0.50)	0.52 ± 0.14 (0.52)	0.51 ± 0.16 (0.50)
Myelin thickness (median)	0.073 ± 0.019 (0.069)	0.073 ± 0.019 (0.070)	0.072 ± 0,016 (0.070)	0.076 ± 0.022 (0.070)	0.079 ± 0.016 (0.079)
g-Ratio (median)	0.75 ± 0.08 (0.77)	0.77 ± 0.07 (0.78)	0.77 ± 0.07 (0.78)	0.77 ± 0.06 (0.77)	0.75 ± 0.07 (0.75)
Conduction velocity (median)	3.51 ± 1.09 (3.34)	3.67 ± 1.14 (3.47)	3.64 ± 1.08 (3.61)	3.66 ± 1.00 (3.73)	3.70 ± 0.91 (3.60)

*Data are mean ± SD. Four rats per group. Diam, diameter; Myel, myelinated; Unmyel, unmyelinated; Ant-l, anterior limb; Post-l, posterior limb; AC, anterior commissure.*

The average myelin thickness of AC (0.086 ± 0.027) and ant-l (0.094 ± 0.027 μm) in chronic hypothyroid rats increased significantly (*P* < 0.05) compared to transient hypothyroid and C rat AC (0.083 ± 0.030) and ant-l (0.089 ± 0.035 μm) (**Table 2**). Chronic hypothyroid average myelin thickness also increased significantly to 0.078 ± 0.018 (*P* < 0.05) in post-l, compared to an average of 0.073 ± 0.018 μm in the other groups; **Figures 9B,E,H,K,N**, **10B,E,H,K,N** and **Table 2**). No significant correlation was found between myelin thickness and inner axon diameter in ant-l and post-l. Notwithstanding, the slope of the ant-l regression function was higher (on average, 3.9 ± 0.6°) than in post-l (on average, 0.9 ± 0.4°; **Figures 9C,F,I,L,O**, **10C,F,I,L,O**).

**FIGURE 11:**
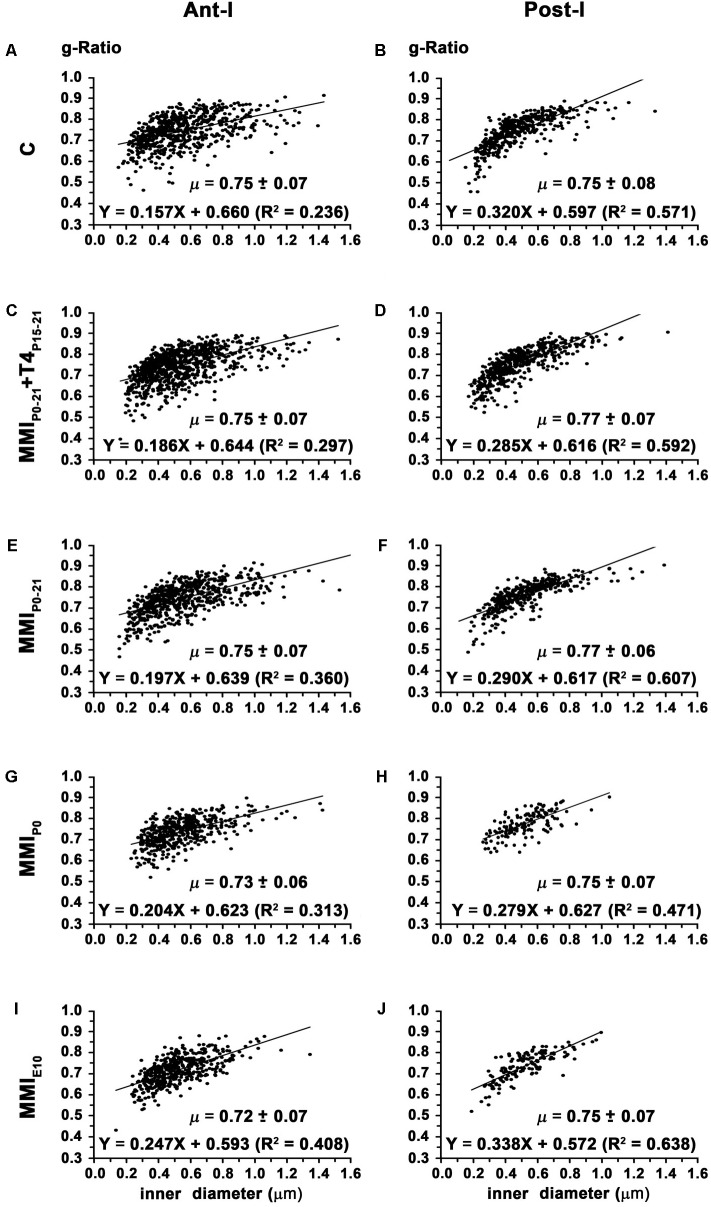
**Correlation between g-ratio and axon diameter in ant-l and post-l at P150.** Plots show the correlation between g-ratio and myelinated axon inner diameter in the ant-l **(A,C,E,G,I)** and post-l **(B,D,F,H,J)** at P150. In ant-l, g-ratio in chronic hypothyroid was lower (*P* < 0.05) than in transient and C rats, while in post-l, it was similar between MMI and C rats. g-Ratio ranged from 0.72 ± 0.07 to 0.75 ± 0.07 in the ant-l, and from 0.75 ± 0.08 to 0.77 ± 0.07 in the post-l. In the ant-l and post-l, the correlation between myelinated axon inner diameter and myelin thickness was low. Mean g-ratio (μ = mean ± SD) and the correlation coefficients (*R*^2^) are indicated.

In chronic hypothyroid AC the average ant-l g-ratio (0.73 ± 0.07) decreased significantly (*P* < 0.05) compared to the 0.75 ± 0.07 ratio in C and transient hypothyroid rats (**Figures 11A,C,E,G,I** and **Table 2**). Similar MMI and C post-l g-ratios (0.76 ± 0.08) were found (**Figures 11B,D,F,H,J** and **Table 2**). The regression function between inner axon diameter and g-ratio was of a similar slope between C and MMI rats, being higher in post-l (16.7°) than in ant-l (11.3°; **Figure 11**).

**FIGURE 12:**
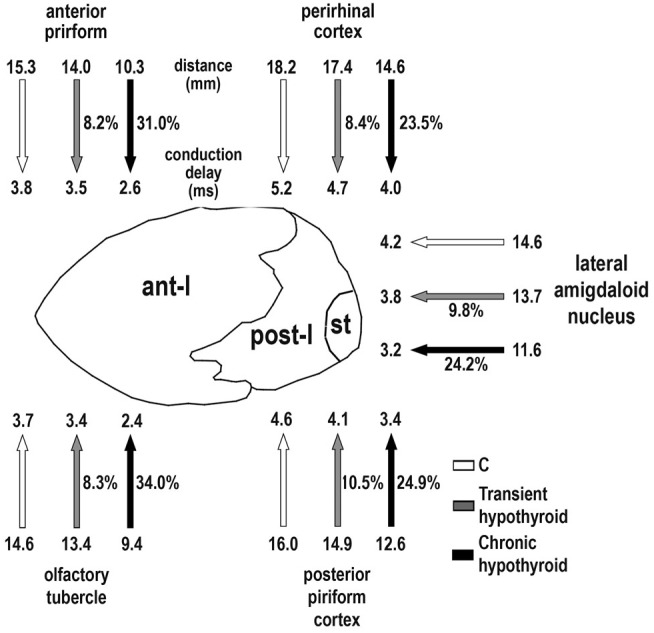
**Conduction delay between AC connected areas.** Cartoon shows main cortical and subcortical areas sending axons through ant-l (olfactory tubercle and anterior piriform cortex) and post-l (perirhinal and posterior piriform cortices, and lateral amygdaloid nucleus). The mean distance (mm) between homotopic contralateral areas (arrow-shaft end) and estimated conduction delay (ms; arrowhead) for C (white arrows), transient (gray arrow) and chronic hypothyroid (black arrows) rats are indicated. In the ant-l, conduction delay between homotopic contralateral areas decreased (arrow-shaft) in transient (on average, 8.2%) and chronic hypothyroid (on average, 32.5%) with respect to C rats. While in post-l, the average decrease was 9.5% in transient and 24.2% in chronic hypothyroid rats. A similar decrease was found in the mean distance between areas. st, stria terminalis.

Conduction velocity was similar between C and MMI rats, being lower in post-l (3.64 ± 1.04 m/s average) than in AC and ant-l (3.94 ± 1.31 m/s average; **Table 2**). The conduction delay between ant-l, and the olfactory tubercle and anterior piriform cortex decreased in transient (on average, 8.2%) and chronic hypothyroid rats (on average, 32.5%) with respect to C (**Figure 12** and **Table 3**). The conduction delay between perirhinal and posterior piriform cortices and lateral amygdaloid nucleus in post-l, also decreased in transient (on average, 9.5%) and chronic hypothyroid rats (on average, 24.2%) with respect to C (**Figure 12** and **Table 3**).

**Table 3:** Distance between areas projecting trough the AC and conduction delay at P150.

		C	MMI_P0-21_+T4_P15-P21_	MMI_P0-21_	MMI_P0_	MMI_E10_
**Ant-l**						
Olfactory tubercle	Distance (mm)	14.6	13.5	13.2	9.8	8.9
	Conduction delay (ms)	3.7 ± 1.4	3.4 ± 1.2	3.3 ± 1.2	2.5 ± 0.7	2.3 ± 0.6
Anterior piriform cortex	Distance (mm)	15.3	14.3	13.7	10.4	10.1
	Conduction delay (ms)	3.8 ± 1.5	3.6 ± 1.3	3.4 ± 1.2	2.7 ± 0.8	2.6 ± 0.7
**Post-l**						
Perirhinal cortex	Distance (mm)	18.2	17.4	17.3	15	14.2
	Conduction delay (ms)	5.2 ± 1.6	4.7 ± 1.5	4.8 ± 1.4	4.1 ± 1.1	3.8 ± 0.9
Posterior piriform cortex	Distance (mm)	16.0	14.9	14.9	12.8	12.4
	Conduction delay (ms)	4.6 ± 1.3	4.1 ± 1.1	4.1 ± 1.1	3.5 ± 0.9	3.4 ± 0.8
Lateral amigdaloid nucleus	Distance (mm)	14.6	13.7	13.7	11.8	11.4
	Conduction delay (ms)	4.2 ± 1.4	3.7 ± 1.3	3.8 ± 1.2	3.2 ± 1.0	3.1 ± 0.8

*Ant-l, anterior limb; Post-l, posterior limb; AC, anterior commissure.*

## Discussion

Our results show that early postnatal transient and chronic hypothyroidism alters AC maturation, which cannot be recovered after delayed T4-treatment. T2r is correlated with myelinated axon number and percentage in the AC of C and MMI rats. Using the corresponding regression functions, myelinated axon number and percentage of MMI rats at P150 was estimated and found to be similar to EM values, showing a decreased percentage and number of myelinated axons in MMI with respect to C rats at P150. g-Ratio was similar between MMI and C rats, but myelinated axon conduction delay decreased in both ant-l and post-l of MMI rats due to their shorter commissural tracts.

### Methodological Considerations: Limitations and Strengths

The major strength of this work is the combination of non-invasive *in vivo* neuroimaging of AC maturation with an *ex vivo* quantitative study of unmyelinated and myelinated axon number and diameter using electron microscopy in the same rat. The quantitative data confirmed delayed AC maturation correlated with T_2_r.

The study was limited to male rats, since gender based differences have been reported in the mid-sagittal AC area of several mammals including rats (Noonan et al., 1998) and humans (Allen and Gorski, 1992). In rats the postero-dorsal region of the medial amygdala and the encapsulated region of the stria terminalis were found to be 97.4 and 85.2% larger, respectively, in male rats, affecting the number of axons connecting through the post-l and stria terminalis (Hines et al., 1992). The use of male pups avoids possible gender based differences in AC mid-sagittal area and unmyelinated and myelinated axon number.

Electron microscopy methodology and measurement taking is particularly time consuming, and as such the EM study sample size was relatively low. The final number of rats per group was limited to four, given that data obtained for MMI_E10_ and C rats at P150 was similar to our estimated values and to quantitative values obtained in previous studies (Gravel et al., 1990; Berbel et al., 1994; Guadaño-Ferraz et al., 1994). Although reduced, this sample size can be considered sufficient to evaluate mean measurement differences between ant-l and post-l and experimental groups.

The fact that T_2_r may not be specific to myelination must be considered. Previous studies suggest that variation in T_2_r reflects changes in histological characteristics of nervous tissue, such as increased myelination and reduction of extracellular space (Chandran et al., 2012; Thiessen et al., 2013; Tsurugizawa et al., 2013; Alonso-Ortiz et al., 2015; Jelescu et al., 2016). In control rats, T_2_r decreased rapidly from P8 to P50, and at a slower pace up to P100 after which it leveled off. The P8-100 decrease is highly correlated to an increased number and percentage of myelinated axons, as seen in previous studies (Berbel et al., 1994). EM studies have shown however, that myelinated axon number in the AC continues to increase after P100 in parallel with an increased number of glial cells and reduced extracellular space (Gravel et al., 1990; Berbel et al., 1994). No relevant changes in T_2_r were observed after P100 in MMI and C rats, suggesting that changes in myelinated axon number are undetectable with currently available MRI technique. This is the case of patients who suffer toxic leucoencephalopathy associated with the use of drugs such as opioids and cocaine (Buttner and Weis, 2006; Alturkustani et al., 2017) in which demyelination of commissural and white matter axons in the cerebral cortex becomes detectable in MRI scans at an advanced stage of the disease (Phan-Ba et al., 2012). Diffusion image measurements of amygdala-lesioned young monkeys are relatively insensitive to white matter changes during recovery (Grayson et al., 2017). This is relevant since early detection by T2w MRI scans of some neurological and psychiatric diseases that affect myelination of cortical axons, and other important factors may not be possible. MRI approaches have been developed to study subtle developmental changes. Enhanced MRI through Mn^2+^ injection (MEMRI; Chan et al., 2012) will provide a useful tool to study commissural connectivity. The diffusion kurtosis image (DKI) is highly sensitive and directionally specific when detecting ultrastructural changes in the brain maturation processes (Cheung et al., 2009). Approaches such as these will add valuable information to the study of developmental ultrastructural changes in white matter and the plasticity of commissural connections in normal and hypothyroid rats.

### Altered Interhemispheric Transfer of Information in MMI Rats

The interhemispheric transfer of information through telencephalic commissures depends on several intrinsic and extrinsic factors, which modulate axon characteristics such as morphology and function. Axon length and inner diameter, myelin thickness and intermodal distances are among the principal factors determining axon function (Rushton, 1951; Waxman, 1975). Importantly, inner diameter and myelin thickness are linked to axon conduction velocity, which combined with axon length relates to conduction delay. These parameters are well established for myelinated axons but remain relatively unknown for unmyelinated axons. Axon diameter has also been related with the size of terminal arbors and boutons of callosal axons in monkeys (Innocenti, 2011; Innocenti and Caminiti, 2017).

In ant-l and post-l, unmyelinated and myelinated axon inner diameter was similar between transient hypothyroid and C rats (**Table 2**), while myelinated axon inner diameter and g-ratio decreased in the ant-l of chronic hypothyroid rats with respect to transient hypothyroid and C rats (**Table 2**). Conduction velocity of myelinated axons was similar in C and MMI rats (although lower in post-l than in AC and ant-l). These data suggest that the transfer of information between hemispheres carried out by unmyelinated and myelinated axons is similar in MMI and C rats. However, myelinated axon number and percentage significantly decreased in transient (on average, 16.9%) and chronic (on average, 55.2%) hypothyroid rats compared to controls, reflecting an increase in the percentage on unmyelinated axons since the total axon number remained similar in C and MMI rats (**Table 1**). The increased number and percentage of unmyelinated axons found in MMI rats results in an overall decrease in conduction velocity, assuming that the conduction velocity of unmyelinated axons is lower than in myelinated, which have a greater inner diameter. While axon diameter and myelination are associated, the length of commissural axons depends on other additional factors. However, since the distance between homotopic areas connected through the AC was less in MMI rats, the corresponding conduction delays estimated from myelinated axon parameters decreased. The study of telencephalic commissures provides additional important information about non-commissural axons (Innocenti, 2017). The decreased number of myelinated axons found in the AC of transient and chronic hypothyroid rats may also reflect abnormal ipsilateral and subcortical connections in cortical areas connected through the AC.

No data has been previously published concerning the mean g-ratio in AC of rats which is similar to those observed in other species, usually ranging between 0.6 and 0.8 (Chomiak and Hu, 2009; Campbell et al., 2017; Innocenti, 2017). However, myelinated axon percentage in control mice at P240 (29.0%) was similar to that found in C rats at P150 (31.1 ± 4.7%) and at P365 (38.2%; Berbel et al., 1994). Myelinated axon diameter (0.53 ± 0.19 μm in mice at P240 vs. 0.55 ± 0.23 in C rats at P150) also remained similar. Although myelin thickness in mice was not calculated by Sturrock (1975), the mean number of myelin lamellae (7.1 ± 3.4) at P240 was similar to that found in C rats at P150 (on average, 7.2; unpublished results), which strongly suggests that myelin thickness was also similar between mice and rats, resulting in similar g-ratio and conduction velocity.

### Altered Behavior in MMI Rats

Four axon bundles merge to form the AC of rats. Two merge in ant-l and contain axons from anterior olfactory nucleus, anterior piriform cortex and olfactory tubercle. Another forms post-l and contains axons from posterior piriform, perirhinal and temporal cortices, and lateral amygdaloid nucleus. The fourth bundle adjacent to post-l is the stria terminalis and contains axons from lateral olfactory tract nucleus, and basolateral, basomedial and cortical nuclei of amygdala (see scheme and references in Guadaño-Ferraz et al., 1994). In primates, commissural projecting areas spread out more than in rodents and the AC also receives axons from virtually the entire temporal lobe, area 13 of orbitofrontal cortex, frontal and temporal subdivisions of pre-piriform cortex, and cortical and deep nuclei of amygdala (Demeter et al., 1990). In humans, projecting areas through the AC also include the inferior part of temporal and occipital lobes, occipital convexity and possibly central fissure and prefrontal convexity (Di Virgilio et al., 1999; Patel et al., 2010). In particular, amygdaloid nuclei play a key role in the modulation of emotion and emotion associated states (Marks, 1987; LeDoux, 2012, 2014). The amygdala receives conditioned stimuli from sensory inputs, meditates defensive reactions (such as freezing, autonomic nervous system activity and hormonal release) by modulating hypothalamic nuclei activity and manages goal-directed actions by modulating ventral striatum activity (LeDoux, 2000, 2012; Davis et al., 2010). Alterations in emotion may lead to different mental illness and have been associated with diseases such as bipolar disorder (LeDoux, 2000; Saxena et al., 2012; Townsend et al., 2013; Janiri et al., 2017) and ADHD (Humphreys et al., 2016). In rats, AC projecting areas are involved mostly in olfactory, anxiety and fear behavior (Amaral, 2003).

Early postnatal and chronic hypothyroid rats showed a marked preference for open arms in the elevated plus maze test (Navarro et al., 2015). Time spent in open arms was 81.1% for MMI_E10-P40_ and 57.0% for MMI_P0-P40_ rats compared to 17.1% for C rats. The number of arm transitions significantly decreased in MMI-treated rats (on average, 4.2 compared to 12.3 transitions in controls). Hypothyroid rats frequently fell from the apparatus, and this behavior was associated with a deterioration in sensory perception (Navarro et al., 2015). Our data show that commissural connections between amygdaloid areas are altered in transient and chronic hypothyroid rats, which may lead to decreased anxiety and an altered perception of danger. The abnormal behavior observed in the elevated plus maze test of chronic hypothyroid rats could be due to both deteriorated perception and alterations of anxiety and fear. The altered maturation of the AC may lead to an increase in impulsive decisions (Bari and Robbins, 2013) which change from adolescence to adulthood (Hunt et al., 2016). Transcriptional T3-regulated genes involved in the juvenile-adult transition of vertebrates have been described (Chatonnet et al., 2015). Delayed perception of dangerous environments and fear in MMI rats suggests a protracted juvenile-like behavior in which arrested AC maturation may play a role.

### Implications for Neurological and Psychiatric Diseases in Humans

In rodents, several hundred genes regulated by T3 at the transcriptional level are known to be involved in basic neurodevelopmental events (Morte et al., 2010; Gil-Ibáñez et al., 2014, 2017; Chatonnet et al., 2015), and some of these genes have also been found mutated in humans suffering neurological and mental diseases (Berbel et al., 2014). Although postnatal cerebral cortex maturation is comparatively longer in humans than in rats (Marín-Padilla, 1978), similarities have been established since most of these T3-regulated genes are involved in evolutionary preserved pathways affecting the establishment of neural connections (Morte et al., 2010; Berbel et al., 2014; Chatonnet et al., 2015). Postnatal hypothyroid rats have been used to mimic human congenital hypothyroidism (Balázs et al., 1969; Morreale de Escobar et al., 1983), and genes involved in neuron-glia adhesion, affecting myelination, have been found to be under-expressed in these rats (Rodríguez-Peña et al., 1993; Rodríguez-Peña, 1999; Barradas et al., 2001; Sharlin et al., 2008). Some are implicated in schizophrenia and bipolar disorders (Tkachev et al., 2003; Santos et al., 2012; Mukherjee et al., 2014) as is the case for myelin basic protein (MBP; Schoonover et al., 2004) and autotaxin (Fuss et al., 1997; Fox et al., 2003; Haas et al., 2004). The Erk1/2 pathway is involved in autotaxin-promoted oligodendrocyte maturation (Liu et al., 2011; Lee and Petratos, 2016) and its expression is decreased in late fetal hypothyroid rats (Berbel et al., 2010). The decreased number and percentage of myelinated axons found in transient and chronic hypothyroid rats might result from not only arrested axon growth but also the decreased proliferation of oligodendrocyte precursors and their maturation due to postnatal thyroid hormone deficiency, albeit transient.

## Conclusion

To conclude, T_2_r increases in the AC of transient and chronic hypothyroid rats and these values correlated with myelinated axon number and percentage in C rats. Quantitative EM data showed that myelinated axon number and percentage decreases in MMI rats at P150, indicating altered connectivity between commissural connected areas in MMI rats. The number and percentage of myelinated axons were similar to those estimated from regression functions obtained from T_2_r. g-Ratio in ant-l and post-l was similar in MMI and C rats, being low in ant-l, because of decreased axon diameter. Conduction delay decreased in MMI rats coinciding with the reduced length of commissural tracts. The AC in MMI rats at P150 showed an impaired axonal maturation that could not be recovered by delayedT4-treatment. Mammals share T3-regulated genes involved in basic signaling pathways during the development and maturation of cortico-cortical connections. Our data may help to a better understanding of the physiopathology of congenital hypothyroidism and calls to attention the increased risk for children suffering postnatal hypothyroidism, albeit transitory, of suffering neurocognitive delay as well as possible psychiatric disorders.

## Author Contributions

PB carried out the conception, design, and draft of the manuscript. FSL, JP-T, SC, and PB contributed to the acquisition, analysis, and interpretation of MRI data. FSL, SG-G, JG-V, and PB contributed to the acquisition, analysis, and interpretation of EM data. M-JO contributed to thyroid hormone determinations. All authors contributed discussing the results and writing specific parts of the manuscript, and gave the final manuscript approval.

## Conflict of Interest Statement

The authors declare that the research was conducted in the absence of any commercial or financial relationships that could be construed as a potential conflict of interest.
