# Supplementary material for: Transient Hypothyroidism During Lactation Arrests Myelination in the Anterior Commissure of Rats. A Magnetic Resonance Image and Electron Microscope Study
Source: Front Neuroanat. 2018 Apr 27;12:31. doi: 10.3389/fnana.2018.00031 (PMC5935182; doi:10.3389/fnana.2018.00031)
Supplement: Supplementary file 1 [file Table_1.PDF]

Supplementary Table 1. Total thyroid hormone concentration in plasma.

Age (days)	C	MMI_{P0-21} $+T4_{P15-21}$	MMI_{P0-21}	MMI_{P0}	MMI_{E10}
			tT4 (ng/ml)		
P15	40.1 ± 5.8	7.8 ± 0.4	8.2 ± 1.0	5.9 ± 0.1	6.9 ± 1.0
P21	54.9 ± 3.5	50.9 ± 2.6	5.9 ± 1.5	5.6 ± 2.7	4.3 ± 0.1
P50	49.0 ± 12.5	41.3 ± 7.8	41.2 ± 3.4	12.7 ± 2.5	13 ± 0.5
			tT3 (ng/ml)		
P15	0.26 ± 0.03	0.06 ± 0.03	0.04 ± 0.03	0.06 ± 0.05	0.04 ± 0.01
P21	0.46 ± 0.05	0.40 ± 0.05	0.04 ± 0.03	0.04 ± 0.01	0.03 ± 0.01
P50	0.38 ± 0.10	0.39 ± 0.06	0.42 ± 0.07	0.05 ± 0.02	0.09 ± 0.01

Data are mean ± SD (n = 6).
