# Supplementary material for: Transient Hypothyroidism During Lactation Arrests Myelination in the Anterior Commissure of Rats. A Magnetic Resonance Image and Electron Microscope Study
Source: Front Neuroanat. 2018 Apr 27;12:31. doi: 10.3389/fnana.2018.00031 (PMC5935182; doi:10.3389/fnana.2018.00031)
Supplement: Supplementary file 2 [file Table_2.PDF]

Supplementary Table 2. T_2r values in the AC at different postnatal ages.

Age (days)	C	MMI_{P0-21} $+T4_{P15-21}$	MMI_{P0-21}	MMI_{P0}	MMI_{E10}
P8	0.59 ± 0.01[a]	0.66 ± 0.02[a]	0.67 ± 0.02[a]	0.66 ± 0.02[a]	0.65 ± 0.02[a]
P15	0.57 ± 0.02 [a]	0.50 ± 0.01[a]	0.52 ± 0.02[a]	0.52 ± 0.03[a]	0.56 ± 0.02[a]
P20	0.47 ± 0.02[b]				
P22	0.42 ± 0.02	0.45 ± 0.01[b]	0.47 ± 0.02[b]	0.45 ± 0.02[b]	0.54 ± 0.02[b]
P24	0.38 ± 0.01				
P27	0.37 ± 0.02				
P30	0.34 ± 0.01	0.35 ± 0.01	0.37 ± 0.02	0.40 ± 0.01[c]	0.46 ± 0.02[b]
P40	0.26 ± 0.02	0.32 ± 0.01	0.35 ± 0.02	0.37 ± 0.02[c]	0.44 ± 0.01[c]
P50	0.27 ± 0.01	0.29 ± 0.01	0.30 ± 0.01	0.31 ± 0.01[c]	0.43 ± 0.01[c]
P60	0.25 ± 0.01	0.27 ± 0.01	0.29 ± 0.01	0.30 ± 0.01	0.38 ± 0.02[c]
P75	0.23 ± 0.01	0.25 ± 0.02	0.28 ± 0.01	0.29 ± 0.01	0.35 ± 0.02
P100	0.23 ± 0.01	0.25 ± 0.01	0.25 ± 0.01	0.29 ± 0.02	0.35 ± 0.02
P125	0.23 ± 0.01	0.26 ± 0.01	0.26 ± 0.01	0.28 ± 0.01	0.35 ± 0.01
P150	0.22 ± 0.01	0.24 ± 0.01	0.25 ± 0.01	0.28 ± 0.02	0.35 ± 0.01
P180	0.20 ± 0.01				0.35 ± 0.01
P365	0.21 ± 0.01				

Values are mean ± SD (n = 8). [a]: the AC is lightly than the adjacent neuropil. [b]: the AC has a similar contrast than the adjacent neuropil. [c]: the AC is lightly darker than the adjacent neuropil. The remaining values correspond to AC darker than the adjacent neuropil.
