# Supplementary material for: Transient Hypothyroidism During Lactation Arrests Myelination in the Anterior Commissure of Rats. A Magnetic Resonance Image and Electron Microscope Study
Source: Front Neuroanat. 2018 Apr 27;12:31. doi: 10.3389/fnana.2018.00031 (PMC5935182; doi:10.3389/fnana.2018.00031)
Supplement: Supplementary file 3 [file Image_1.PDF]

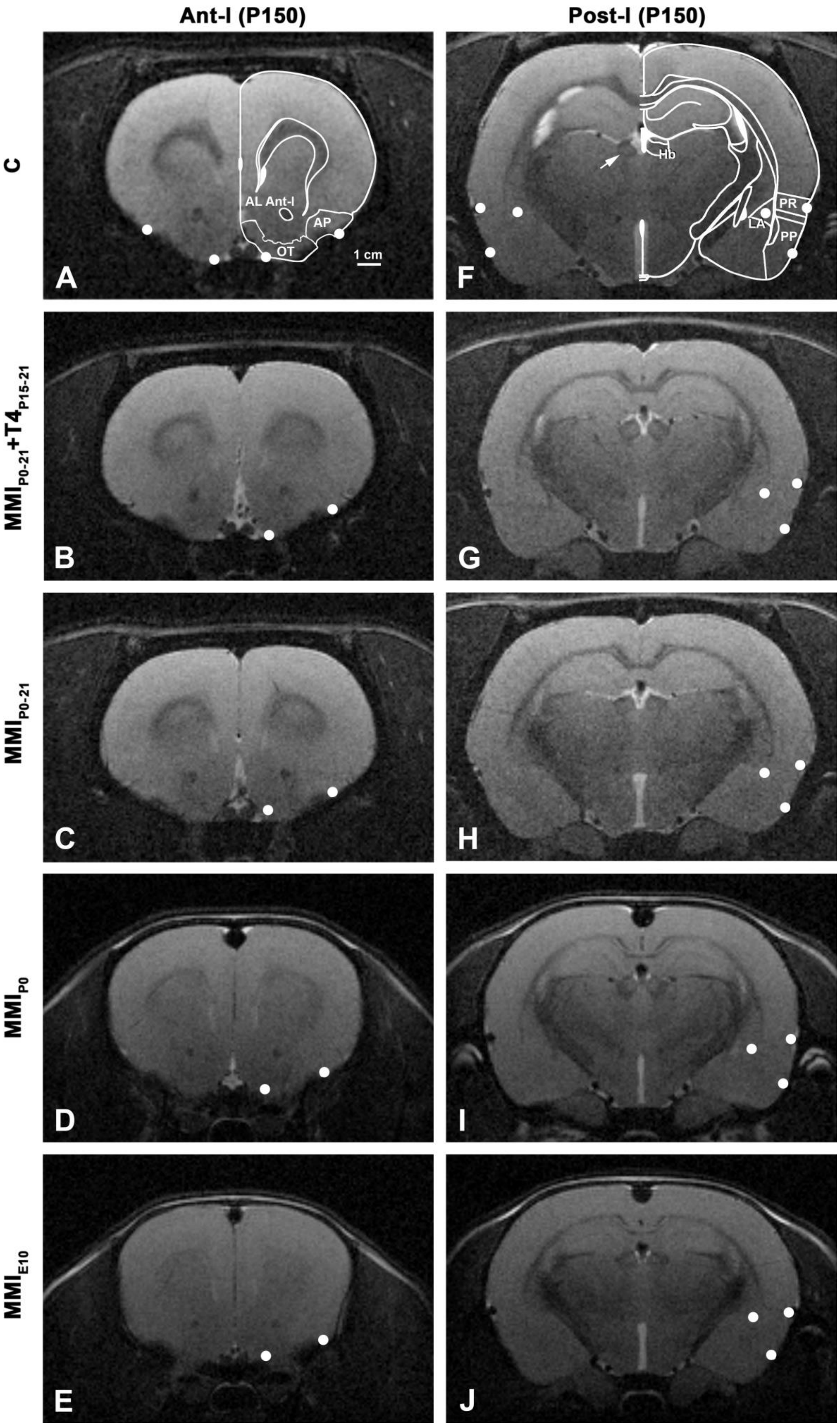

Ant-I (P150)
Post-I (P150)
C
MMI P0-21 +T4 P15-21
MMI P0-21
MMI P0
MMI E10
AL Ant-I
AP
OT
1 cm
Hb
PR
LA
PP
A
B
C
D
E
F
G
H
I
J

Supplementary figure 1. MRI images showing ROIs of telencephalic areas projecting through the AC. T2-weighted images (**A-J)** of coronal sections of MMI and C rats at P150 show selected commissural projecting areas. Outlines (**A,B)** show the boundaries of the selected areas in C rats, according to Patxinos et al. (2015). At 1.89 mm from Bregma in C rats (A), the antero-lateral (AL) branch of the ant-l begins to appear transversally cut. This has been taken has a landmark for MMI rats. At this level, olfactory tubercle (OT) and anterior piriform (AP) cortex can be seen in C (**A**) as well as in MMI (**B-E**) rats. At -3.36 mm from Bregma in C rats **(B)**, the lateral and medial habenular (Hb) nuclei appear darker than the adjacent neuropil (arrow). This has been taken has a landmark for MMI rats. At this level, the perirhinal (PR) and posterior piriform (PP) cortices and lateral amygdaloid (LA) nucleus can be seen in C (**F**) as well as in MMI (**G-J**) rats. Contralateral distances were measured between homotopic regions of interest (ROIs; dots) located at mid-zones of pial surfaces of selected areas and the central zones of LA. Same scale for all figures.
